# Risk Factors for Disseminated Histoplasmosis in a Cohort of HIV-Infected Patients in French Guiana

**DOI:** 10.1371/journal.pntd.0002638

**Published:** 2014-01-30

**Authors:** Mathieu Nacher, Antoine Adenis, Denis Blanchet, Vincent Vantilcke, Magalie Demar, Célia Basurko, Emilie Gaubert-Maréchal, Julie Dufour, Christine Aznar, Bernard Carme, Pierre Couppié

**Affiliations:** 1 Centre d'Investigation Clinique, Epidémiologie Clinique Antilles Guyane (CIC EC CIE802), Centre Hospitalier de Cayenne, Cayenne, French Guiana; 2 COREVIH Guyane, CH de Cayenne, Cayenne, French Guiana; 3 Equipe EA 3593 Epidemiologie des Parasitoses et Mycoses Tropicales, Université Antilles Guyane, Campus Saint Denis, Cayenne, French Guiana; 4 Laboratoire Hospitalo Universitaire de Parasitologie Mycologie, CH de Cayenne, Cayenne, French Guiana; 5 Service de Médecine, Centre Hospitalier de l'Ouest Guyanais, Saint Laurent du Maroni, French Guiana; 6 Unité des Maladies Infectieuses et Tropicales, Centre Hospitalier de Cayenne, Cayenne, French Guiana; 7 Service Dermatologie, CH de Cayenne, Cayenne, French Guiana; Fundação Oswaldo Cruz, Brazil, United States of America

## Abstract

Disseminated histoplasmosis is the first AIDS-defining infection in French Guiana. A retrospective cohort study studied predictive factors of disseminated histoplasmosis in HIV-infected patients between 1996 and 2008. Cox proportional hazards models were used. The variables studied were age, sex, last CD4/CD8 count, CD4 nadir, herpes or pneumocystosis, cotrimoxazole and fluconazole use, antiretroviral treatment and the notion of recent initiation of HAART. A total of 1404 patients were followed for 6833 person-years. The variables independently associated with increased incidence of disseminated histoplasmosis were CD4 count<50 per mm3, CD4 count between 50 and 200 per mm3, a CD4 nadir <50 per mm3, CD8 count in the lowest quartile, herpes infection, and recent antiretroviral treatment initiation (less than 6 months). The variables associated with decreased incidence of histoplasmosis were antiretroviral treatment for more than 6 months, fluconazole treatment, and pneumocystosis. There were 13.5% of deaths at 1 month, 17.5% at 3 months, and 22.5% at 6 months after the date of diagnosis of histoplasmosis. The most important predictive factors for death within 6 months of diagnosis were CD4 counts and antiretroviral treatment. The present study did not study environmental/occupational factors but provides predictive factors for disseminated histoplasmosis and its outcome in HIV patients in an Amazonian environment during the HAART era.

## Author Summary

Disseminated histoplasmosis is the first AIDS-related disease in French Guiana, and probably in the Amazonian area. In order to determine the factors that are associated with histoplasmosis, a retrospective looked at a cohort of HIV-infected patients between 1996 and 2008. Multiple models were used to study the relation of age, sex, last CD4/CD8 count, CD4 nadir, herpes or pneumocystosis, cotrimoxazole and fluconazole use, antiretroviral treatment and the notion of recent initiation of antiretroviral treatment with the occurrence of disseminated histoplasmosis. A total of 1404 patients were followed for 6833 person-years. The variables independently associated with the incidence of disseminated histoplasmosis were low CD4 counts, the lowest CD4 counts were most at risk; Patients with the lowest CD8 counts were also at increased risk; Antiretroviral treatment was generally associated with lower histoplasmosis incidence, but for the first 6 months following antiretroviral treatment initiation there was a transient period of increased risk of diagnosing histoplasmosis; Herpes was also associated with more histoplasmosis; Pneumocystosis and Fluconazole treatment were negatively associated with histoplasmosis. Of 156 patients with histoplasmosis, there were 13.5% of deaths at 1 month, 17.5% at 3 months, and 22.5% at 6 months after the date of diagnosis of histoplasmosis. The most important predictive factors for death within 6 months of diagnosis were low CD4 counts and no antiretroviral treatment. The present study did not study environmental/occupational factors but provides predictive factors for disseminated histoplasmosis and its outcome in HIV patients in an Amazonian environment during the HAART era. These results are useful to guide clinicians working in an area where this diagnosis is often overlooked.

## Introduction

*Histoplasma capsulatum var. capsulatum* (HC) is found throughout the world, but there are great differences in the levels of endemicity [1]. On the South American continent, histoplasmin sensitivity studies showed proportions of the population with positive tests ranging from 7% to nearly 90%. On the Guiana Shield, the proportion of persons with positive tests is around 30%. Microconidia and mycelial forms of HC are present in the soil and aerial dispersion exposes persons to inhale these infective forms. In immunosupressed persons, HC yeasts then disseminate to various organs through phagocytes, notably macrophages where they can survive for lack of cellular activation by a robust proinflammatory immune response. Disseminated histoplasmosis has been an AIDS defining infection of HIV-infected patients since 1987 [2]. The disease may follow the resurgence of a previous infection due to immunodepression, or it may be newly acquired [3]. It often affects the most severely immunosupressed patients, and when untreated, usually leads to death. Approximately 10% of patients present with a septic shock-like syndrome [4] with high mortality. Even in the absence of initial shock, a significant proportion of cases of disseminated histoplasmosis (ranging from 22% to 47%) are severe and have a poor prognosis [5].

There have been few prospective studies on the predictive factors of disseminated histoplasmosis in HIV patients, mostly in the United States of America [3], [6]. Environmental and occupational aspects, and the patient characteristics that were associated with increased risk have been studied in the 1990's. In the Amazonian area and in the Guianas, some data about histoplasmosis suggest that this is a major –but dramatically underdiagnosed- AIDS defining illness [7], [8]. In the absence of diagnosis, the problem remains invisible, and therefore, in some endemic countries, standard drugs such as itraconazole are not available. There is thus a need to describe its epidemiology and to raise the awareness of clinicians and decision makers about this disease. The objective of the present study was thus to describe the predictive factors of disseminated histoplasmosis in a cohort of HIV-infected patients followed in French Guiana and to determine predictors of death.

## Methods

### Patients

HIV positive patients followed in Cayenne, Kourou, and Saint Laurent du Maroni Hospitals between January 1^st^ 1996 and October 31^st^ 2008 were enrolled in the French Hospital Database for HIV (FHDH). The data is entered in the FHDH database by trained technicians from the medical records. Diagnoses were coded according to the 10^th^ international classification of diseases. Occupational and environmental data were not available in the FHDH. The variables usually used as prognostic factors [5] (LDH, haemoglobin, platelet counts, ferritine, liver enzymes, creatinine, albumine, symptoms) were not available in the database which was created to follow broader trends.

### Diagnosis of histoplasmosis

The diagnosis of histoplasmosis was performed by direct examination using May Grünvald Giemsa staining and culture of tissue and fluid samples for up to 3 months. Primary prophylaxis for disseminated histoplasmosis is not given.

### HIV care in French Guiana

All HIV patients in French Guiana can receive free antiretroviral treatments (including the most recent drugs) regardless of their origin or socio-economic level. Imagery, Viral loads, CD4 counts and genotyping and antiretroviral concentration measurements are available for routine care.

### Study design and statistical analysis

In this retrospective cohort study, incidence rates were obtained. Kaplan Meier curves were used to visualize the differences in the incidence of histoplasmosis between CD4 strata and between different CD4 and CD8 strata. Single failure multiple Cox proportional hazards models were used to evaluate the adjusted relationship between failure and a set of explanatory variables. Right censoring occurred after the last visit. For the first model, including 1404 patients, the failure event was a first episode of disseminated histoplasmosis. The main explanatory variables were for the time independent variables: sex, age, and a nadir of CD4 count<50/mm3, a prior history of herpes or pneumocystosis [6]; for the time dependent variables: last available CD4 cell count at the time of the visit (categorized 0–50, 51–200, 201–350, 350–500, and >500 cells per mm^3^), last available CD8 cell count at the time of the visit (dichotomous variable corresponding to CD8 values within the lowest quartile or not), cotrimoxazole and fluconazole use, the presence or absence of HAART and the notion of recent initiation of HAART (<6 months) [9]. A variable reflecting the annual frequency of visits was also added to the models. First the crude hazard ratios were obtained for each predictor, afterwards a multiple model with the relevant variables was constructed. Confounding was considered when the difference between crude and adjusted hazard ratios exceeded 20%. Different interaction terms were created between explanatory variables and added in succession to the full model and removed when non significant. Overall, none of the interaction terms was retained in the final model. The proportionality of the hazard functions was determined using Schoenfeld and scaled Schoenfeld residuals and the global proportional hazards test.

A second model was constructed in a subgroup of 156 patients with disseminated histoplasmosis with death within 6 months of diagnosis as a failure event and CD4 count, CD8 counts, age, sex and antiretroviral treatment as explanatory variables. Other treatments, such as fluconazole and cotrimoxazole were also explored.

The significance level was 0.05. The Data were analyzed with STATA 12.0 (College Station, Texas, USA).

### Ethics statement

Patients included in the FHDH gave written informed consent to the use of their data for the study. Their identity was encrypted before the data was sent to the Ministry of Health and the Institut National de la Recherche Médicale (INSERM) which centralize data from Regional Coordination for the fight against HIV (COREVIH) throughout France. This cohort is approved by the Commission Nationale Informatique et Libertés (CNIL) since Nov 27^th^ 1991 and has led to numerous international publications.

## Results

### Follow up

A total of 1404 patients were included. This amounted to 30838 records and 6833 years at risk. There were 141 first episodes of disseminated histoplasmosis observed. The average time at risk was 4.04 years. The general characteristics of the patients at inclusion are shown in table 1.

**Table 1:** General characteristics of the patients included in the cohort.

Age group (years)	Female N (%)	Male N (%)	French nationality N (%)	CDC Stage C* (%)
<20	91 (86.7)	14 (13.3)	18 (26.8)	9.5
[20–30[	387 (68.7)	176 (31.2)	71 (15.6)	12.9
[30–40[	407 (51.4)	385 (48.6)	148 (24)	24.4
[40–50[	211 (43)	280 (57)	85 (21.7)	27.7
[50–60[	92 (37.4)	154 (62.6)	66 (33.3)	28.5
>60	46 (36.5)	80 (63.5)	43 (45.7)	27.8

* 1993 revised classification system for HIV infection and expanded surveillance case definition for AIDS among adolescents and adults. *MMWR Recomm Rep* **41** (RR-17): 1–19. December 1992.

### Incidence

The overall incidence rate for a first episode of disseminated histoplasmosis was 1.41 per 100 person years.

**Figure 1:**
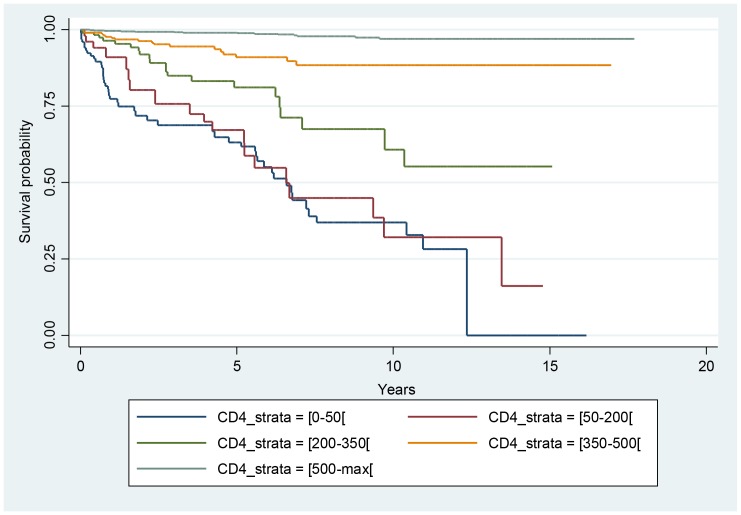
Incidence of a first episode of histoplasmosis stratified by CD4 count. The Y axis represents the percentage of persons that have never had histoplasmosis.

**Figure 2:**
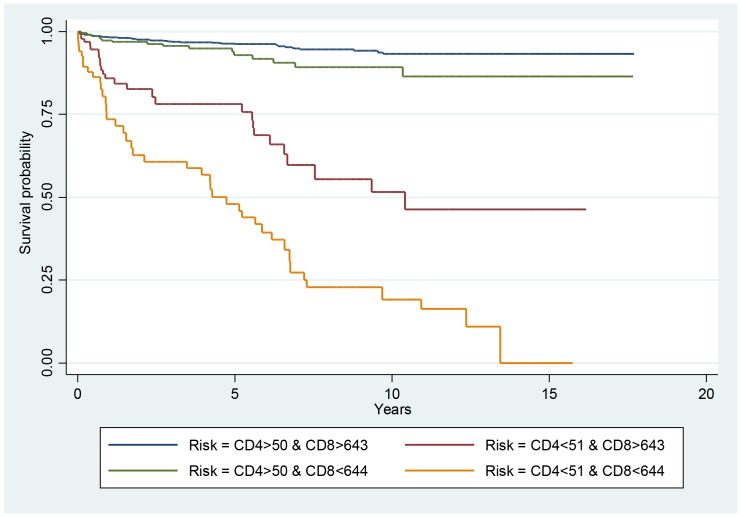
Incidence of a first episode of histoplasmosis stratified by different combinations of CD4 and CD8 counts: The Y axis represents the percentage of persons that have never had histoplasmosis.

Figure 1 shows the Kaplan Meier curves for different CD4 strata, with a marked increase of the incidence of histoplasmosis in patients with CD4 counts <50 per mm^3^. Patients with both CD4 counts <50 per mm3 and CD8 counts under 643 had the highest risk of histoplasmosis (Fig. 2).

Table 2 shows the variables associated with disseminated histoplasmosis in the HIV cohort of French Guiana.

**Table 2:** Independent predictors of a first episode of disseminated histoplasmosis in a cohort of HIV-infected patients in French Guiana: 1996–2008.

Variable	Incidence rate (per 100 person-years)	Crude hazard ratio	Adjusted hazard ratio* (95% CI)	*P*
**Age (years)**				
18–30	0.9	1	1	
31–40	2.1	2.7 (1.5–4.6)	1 (0.5–1.9)	0.9
41–60	1.4	1.8 (1–3.2)	0.8 (0.4–1.6)	0.6
61-max	1.2	1.5 (0.7–3.7)	0.9 (0.3–2.6)	0.8
**Sex**				
Men	2.1	1.4 (1.2–1.7)	1.4 (1.1–1.7)	0.004
Women	1			
**CD4 count** (per mm3)				
[0–50[	11.8	118.8 (29–485)	47.2 (5.8–380)	<0.001
[50–200[	2.4	23.8 (5.7–98.6)	16.9 (2.2–128)	0.006
[200–350[	0.6	6.1 (1.4–27)	7.1 (0.9–55)	0.06
[350–500[	0.1	1.1 (0.1–7.8)	1.8 (0.16–20)	0.6
[500-max]	0.1	1	1	
**CD4 nadir <50/mm3**				
Yes	4.7	2.1 (1.1–3.9)	1.9 (1–3.6)	0.05
No	0.6			
**CD8 count in the lowest quartile** (<643 per mm3)				
Yes	3.5	1.9 (1.3–2.7)	1.8 (1.2–2.9)	0.008
No	0.7			
**Antiretroviral treatment**				
Yes	0.7	0.4 (0.2–0.5)	0.2 (0.1–0.4)	<0.001
No	2.4			
**First six months of antiretroviral treatment**				
Yes	11.1	2.8 (2–4)	2.4 (1.1–5)	0.01
No	2.3	0.7 (0.5–0.9)	0.5 (0.2–0.9)	0.03
No treatment	3.9	1		
**History of herpes**				
Yes	17.1	10.3 (5.7–18.6)	6.4 (3.1–13.2)	<0.001
No	1.4			
**History of Pneumocystosis**				
Yes	8.7	4.3 (1.8–10.6)	0.1 (0.0–0.5)	0.003
No	1.4			

* Cox multiple model in HIV positive patients with first episode of disseminated histoplasmosis as failure event. Model with 1404 subjects and 94 single failures.

The incidence rate increased proportionally to the level of CD4 decline. Table 1 also shows that patients that had a CD4 nadir <50 had a greater risk of disseminated histoplasmosis. Patients in the lowest CD8 quartile had an increased incidence of disseminated histoplasmosis. There were important differences between the crude and adjusted hazard ratios suggesting confounding, notably by the CD4 nadir.

Antiretroviral treatment was associated with protection from histoplasmosis (models with different antiretroviral classes did not show any difference between classes, data not shown). However, the first 6 months following antiretroviral treatment initiation were a period of increased risk of diagnosing histoplasmosis (table 2).

After adjustments in Cox multiple models, cotrimoxazole was not associated with any protection from disseminated histoplasmosis while herpes was associated with an increased risk of disseminated histoplasmosis (table 2). When looking at the temporal relation between herpes and histoplasmosis 11 herpes cases (50%) were simultaneous with disseminated histoplasmosis, and 4 cases (18%) occured within six months before the diagnosis of disseminated histoplasmosis. There was a notable difference between the crude and adjusted hazards reflecting confounding.

On the contrary, after controlling for CD4 count, and cotrimoxazole use, a history of pneumocystosis (but not toxoplasmosis) was independently associated with a decreased risk of disseminated histoplasmosis. However, there were 3 simultaneous cases of pneumocystosis and disseminated histoplasmosis. Adjustments for the annual frequency of visits did not change the observed association between pneumocystosis and protection from histoplasmosis and thus, for the sake of parsimony, this variable was excluded from the final model. No other opportunistic infection was associated with histoplasmosis in the multiple single failure model. It is of note that tuberculosis was associated with disseminated histoplasmosis in an analysis with a single covariable and in a multiple failure model (but not in the single failure model) multiple model with the same covariables (Adjusted Hazard Ratio = 2.3 (95%CI = 1.1–4.6, *P* = 0.016). Altogether, 12% of first histoplasmosis cases had a concomitant opportunistic infection.

After adjustments in Cox multiple models, fluconazole treatment was associated with a reduction of the incidence of histoplasmosis. The crude incidence rate seemed higher in those receiving fluconazole (Table 3), but this reflected the underlying immunosuppression. When looking at the incidence in the <50 CD4 per mm3 strata, those having received fluconazole had a lower incidence of histoplasmosis than those who did not receive it (6.5 per 100 person-years vs 12.9 per 100 person-years, respectively). Unsurprisingly, curative treatments such as itraconazole, amphotericine B, or liposomal amphotericine were positively associated with histoplasmosis because they were initiated when the diagnosis of histoplasmosis was made, before that they were exceptionally prescribed. They were thus not included in the predictive models. Interaction terms between CD8 and CD4 counts were created but were not significant and thus removed from the Cox model.

**Table 3:** Azoles and incidence of a first episode of histoplasmosis.

	Incidence rate	Crude hazard ratio	Adjusted hazard ratio* (95% CI)	*P*
	(per 100 person-years)			
**Oral Fluconazole**				
Yes	4.3	2.8 (1.5–5.3)	0.4 (0.1–1)	0.05
No	1.4			
**Cotrimoxazole prophylaxis**				
Yes	3.9	4.9 (3.5–6.8)	1.3 (0.8–2.1)	0.2
No	0.8			

* Cox multiple model in HIV positive patients with first episode of disseminated histoplasmosis as failure event. Model with 1404 subjects and 94 single failures.

### Mortality

Of 156 patients with disseminated histoplasmosis, there were 13.5% of deaths at 1 month, 17.5% at 3 months, and 22.5% at 6 months after the date of diagnosis of histoplasmosis. The factors associated with death are shown in table 4. Among the available variables, the most important predictive factors were CD4 counts and antiretroviral treatment. A history of oral fluconazole or cotrimoxazole treatment prior to disseminated histoplasmosis was not associated with any significant differences in mortality.

**Table 4:** Predictors of death within 6 months in HIV infected patients with disseminated histoplasmosis in French Guiana: 1996–2008.

Variables	Crude hazard ratio (95% CI)	Adjusted hazard ratio* (95% CI)	*P*
Male gender	1.9 (1.3–2.7)	1.9 (1.2–3)	0.005
Antiretroviral treatment	0.1 (0.0–0.5)	0.2 (0.0–0.5)	0.003
CD8 count in the lowest quartile (<643 per mm3)	9.6 (4–22.6)	4.3 (1.1–7.5)	0.002
CD4<50 per mm3	30 (13–67)	14.6 (5.7–37)	<0.001

* Cox model in HIV positive patients with disseminated histoplasmosis with death at 6 months as failure event adjusted for sex, antiretroviral treatment, CD4 count (below 50/mm3 or not) and CD8 count (below first quartile or not). Oral fluconazole or cotrimoxazole were not significantly linked to outcome, and thus removed from the final model with 156 subjects and 28 failures.

## Discussion

The present results show a markedly lower incidence of histoplasmosis in HIV infected patients in French Guiana (1.41 per 100 person years) than in the USA (4.7% per year) [3]. First, while the American study took place before highly active antiretroviral therapy (HAART) was available, the present study covered a period where over 80% of patients received HAART, which may have globally increased the level of immunity and reduced the incidence of disseminated histoplasmosis. In addition, histoplasmin skin test positivity studies suggest that histoplasma is much more Frequent in the middle west (60–90%) [10] than in French Guiana where, on the basis of local studies [11] and studies in neighbouring countries [8], [12], it is estimated to be around 30%.

HIV-positive men had a higher risk of histoplasmosis, and of death within 6 months if they had histoplasmosis, both of which had not been observed in the studies in the USA [3], [6]. However, histoplasmin skin test studies have shown a slight male bias [10], which presumably reflects the gender differences regarding their environmental and occupational niches. Previous studies have shown that males had a higher AIDS mortality in French Guiana [13] and elsewhere [14]; The present observation may also result from the same contextual determinants. The patients with the lowest CD4 counts were both at increased risk of histoplasmosis and death within six months for patients with histoplasmosis. In addition to CD4 counts around the time of diagnosis, the CD4 nadir was also an independent predictor of disseminated histoplasmosis as was demonstrated for other indicators of HIV disease progression [15].

A less straightforward finding was the observation that CD8 counts in the lowest quartile were independently associated with the incidence of histoplasmosis, and death within six months for patients with histoplasmosis. Some studies have shown that CD8 cell depletion affected the course of fungal infections [16], [17]. CD8 depletion could have resulted from the dissemination of the fungal pathogen, or from HIV itself [18], [19], [20]. CD8 counts often have a murky significance for clinicians. The present finding possibly offers a coarse glimpse on the nature of the immune response, but in practice seems unlikely to be very helpful for clinicians.

As observed elsewhere, after adjustments in Cox multiple models, antiretroviral treatment was independently associated with protection from disseminated histoplasmosis [6]. There was, as described before [9], a transient increase in the incidence within 6 months of antiretroviral treatment initiation presumably reflecting a surge of diagnoses following immune reconstitution. Oral fluconazole, although it is not as effective as itraconazole against HC, was also associated with decreased incidence of disseminated histoplasmosis but not with differences in mortality within 6 months of diagnosis. This is consistent with some previous observations [6] but not with other studies in the USA that did not observe any benefits of fluconazole in preventing histoplasmosis [3], [21], [22]. However, the present study involved a relatively large number of patients and may have had more power to detect moderate protective effects.

In 2001, Hajjeh *et al*. reported that pneumocystosis was associated with a lower risk of histoplasmosis and that herpes was associated with a poor outcome of histoplasmosis [6]. The present study also found that pneumocystosis was associated with a lower risk of histoplasmosis and found that patients with a history of herpes had an increased risk of histoplasmosis. The explanation for this is not clear. Perhaps pneumocystosis occurs earlier in the course of the HIV infection and may lead to initiate a better follow up and treatment, thereby preventing further loss of CD4 cells and the risk of severe immunodepression and disseminated histoplasmosis. Cotrimoxazole was not associated with a modified incidence of first episodes of disseminated histoplasmosis. Cerebral toxoplasmosis, which occurs at similar levels of immunodepression and often leads to similar prophylactic treatment, was not related to the incidence of disseminated histoplasmosis, or to its outcome as reported elsewhere [6]. Finally, pneumocystosis could influence the bronchial mucosal defences against *Histoplasma*, but this broad speculation should be tested in prospective studies.

The association of herpes with histoplasmosis may reflect the fact that clinical herpes lesions were triggered by latent histoplasmosis, or that clinical herpes reflected growing immunodepression. The single failure multiple Cox model using only the first episode of histoplasmosis did not show any link between a history of tuberculosis and histoplasmosis and was removed from the final model. However, the multiple failure model, showing relapses or reinfections showed that tuberculosis, as reported in the literature [5], was associated with disseminated histoplasmosis.

Previous studies in the American middle west had shown the occupational and environmental risk factors of HIV-associated histoplasmosis [3], [6] and some variables such as CD4 count [6], and past medical history and treatments [3]. The data collected for the FHDH does not include environmental and occupational data. Therefore, the present study could not explore these risk factors in the context of French Guiana. Most of the usual prognostic factors are not recorded in the FHDH, a cohort that does not go into fine clinical and biological detail. Therefore, the variables used are not of major importance for clinicians to identify prognostic elements influencing treatment [23]. However, the main objective of the present study was not to study prognosis. Despite these limitations, the present study provides additional information using longitudinal data from HIV patients in an Amazonian environment during the HAART era. The harzard ratios in the single models were often confounded, mostly by the CD4 count, as shown by the difference with the multiple models.

In conclusion, immunological factors such as low CD4 count, low CD8 count, low CD4 counts at the Nadir, the absence of antiretroviral treatment and/or oral fluconazole, and male gender were associated with an increased risk of histoplasmosis. Regarding mortality, low CD4 count, low CD8 count, absence of antiretroviral treatment, male gender and an age under 30 years were associated with death within 6 months.
